# Self-Medication in Individuals With Depression and Symptoms of Depression in the European Union: Prevalence and Associated Factors

**DOI:** 10.1155/da/4661541

**Published:** 2025-08-15

**Authors:** Spencer Yeamans, Pilar Carrasco-Garrido, Valentín Hernández-Barrera, Ángel Gil-De-Miguel

**Affiliations:** Department of Medical Specialties and Public Health, Universidad Rey Juan Carlos, Alcorcón, Madrid, Spain

**Keywords:** EHIS, European Health Interview Survey, gender, mental health, nonprescription, over-the-counter, sex

## Abstract

Depression is a growing public health problem in the European Union (EU), with many individuals turning to self-medication (SM) to manage their symptoms. This cross-sectional study uses data from the third wave of the European Health Interview Survey (EHIS; 2018–2020) to examine the prevalence and determinants of SM among people with recognized depression and depressive symptoms. A total of 25,701 respondents were analyzed. Prevalence of SM among individuals with recognized depression and symptoms of depression in the EU is 38.46% in men and 46.84% in women, varying considerably between countries. An important finding of this study is the impact of medication availability, with access to over-the-counter (OTC) medications outside of pharmacies nearly doubling SM likelihood (adjusted odds ratio [AOR] = 1.98). Additionally, the results reveal marked differences in how these men and women self-medicate. Specifically, women are more likely to self-medicate with depressive symptoms versus recognized depression (AOR = 1.28), whereas the opposite is observed in men (AOR = 0.69). Among women, younger age groups with depression symptoms are particularly likely to self-medicate (15–24 AOR = 1.60; 25–44 AOR = 1.93) and the results reinforce education as a strong predictor of SM (higher education vs. no education AOR = 5.63). Visits to medical/surgical specialists are also linked to SM in women (AOR = 1.32). This study also highlights potentially concerning relationships between SM and alcohol use in men with recognized depression (AOR = 1.42) and prescribed medicine (AOR = 1.68). Differences are also observable in the effect of employment on SM (AOR = 1.45) in men with depression symptoms and women with recognized depression. In contrast, physical activity (PA; high vs. low AOR = 1.32) and healthcare barriers (distance/transportation issues AOR = 1.89 in women; AOR = 1.55 in men, inability to afford care AOR = 1.38) display similar positive associations in men and women. Taken together, these findings underscore the complex and multifaceted nature of SM and point to potential gaps in depression care across the EU, emphasizing the need for gender-sensitive public health strategies and a closer look at OTC medication access.

## 1. Introduction

Depression is a public health problem with the largest global burden of all mental disorders and is currently on the rise in the European Union (EU) [1, 2]. Varying across EU member states, the total prevalence of depression in the EU is under debate, ranging from 2.10% to 6.54% depending on the employed measurement tool [2, 3]. To deal with their symptomology, the self-medication (SM) hypothesis states that those suffering from psychological ailments such as depression may turn to the consumption of substances such as medication [4]. Undeniably, the link between depression and the consumption of medications, including psychopharmaceuticals but also opioids, with and without prescription, is well-established [5, 6]. A study in the United States working with university students found a connection between nonmedical use of prescription medicine (NMUP) and depression with respect to opioids, stimulants, sedatives, and antidepressants [7].

However, little research has investigated the link between depression and broader SM habits in general population. Though definitions vary, SM can be best defined as the use of over-the-counter (OTC) and prescription medications, herbal products, home-made remedies, nutritional supplements, and vitamins without strict adherence to authorized health care professionals´ instruction regarding indication, dose, and duration of treatment [8]. Like depression, SM is increasingly frequent [8], with reports emerging which cite eroding consumer trust in pharmacists and increasing information access as driving causes [9, 10]. Research from the United States found that the average consumer makes 26 trips annually to purchase OTC products, while only performing three doctors' visits during the same time frame [11].

Undoubtedly, responsible SM forms a key part of public health strategy and provides numerous benefits, including time and cost savings, quality of life improvements, and reduced strain on health care systems [12]. Concerningly, however, an Australian study found that less than 20% of patients reported often following dosage, frequency, and duration directions for nonprescription medicines [13]. Despite the fact that 71% of participants in a Scottish study perceived no risk from nonprescription medicines [14], irresponsible SM poses a multitude of risks. These include delayed disease diagnoses, antimicrobial resistance, comorbidities, drug–drug interactions, adverse drug reactions, misuse, and addiction [12]. As a result, understanding trends in SM is essential to broader public health. A previous study utilizing a 2-week sampling window established SM prevalence in the EU at 34.3% and discovered substantial SM prevalence variability between EU countries [15]. Additionally, this study established several socioeconomic, demographic, lifestyle, and health factors as determinants of SM in the EU.

Nonetheless, a number of questions remain surrounding the relationship between SM and vulnerable populations such as those with depression. Consequently, the goal of this study is to analyze SM prevalence and its associated factors in individuals ages 15 and older in the EU with recognized depression and symptoms of depression.

## 2. Materials and Methods

### 2.1. Data Source and Study Population

This observational study utilizes anonymized data from the third wave of the cross-sectional European Health Interview Surveys (EHISs) [16, 17]. Surveys were conducted between January 2018 and September 2020 on a broad representative sample of the population, consisting of the noninstitutionalized subjects aged 15 and over residing in EU countries. Twenty-six of the 27 EU member states are represented in this study, as France's data was unavailable via Eurostat. Results are comparable between countries due to the use of a common regulatory framework, including common variable definitions and conceptual guidelines, protocols for survey administration, and a standardized questionnaire, translated as needed to the local population. The total weighted sample size for this study is 25,701.

### 2.2. Study Measures

The dependent variable in this study is the dichotomous answer (yes/no) to the question “During the past 2 weeks, have you used any medicines or herbal medicines or vitamins not prescribed by a doctor?” excluding contraceptive pills or hormones used solely for contraception. In the construction of the dependent variable, we eliminated proxy answers to the dependent variable question for homogenization purposes.

To better analyze relationship between depression and SM, the study sample is partitioned into two mutually exclusive groups. The first group, labeled recognized depression, contains those who marked “yes” to the depression component of the question “During the past 12 months, have you had any of the following diseases or conditions?” The second group, labeled symptoms of depression, consists of those who do not enter into the previous grouping, but scored 10 points or more on the eight questions composing the Patient Health Questionaire-8 (PHQ-8). The PHQ-8 is Likert-type scaled tool validated for screening for depression in the EU [18, 19]. The symptoms of depression group does not contain participants from Spain, as the questions used to construct the PHQ-8 were excluded from Spain´s dataset. Results for both of these groups are also divided by sex.

This study also includes demographic, socioeconomic, lifestyle, and health independent variables. Demographic variables consist of age (separated into five categories: 15–24, 25–44, 45–64, 65–74, 75+), sex, marital status and living situation (single, married or living with partner, divorced, or widowed), degree of urbanization (cities, towns/suburbs, or rural), and nationality. Nationality has three potential responses: native-born, born in another EU member state, or born in a non-EU country. Due to anonymization practices, Malta´s nationality data was excluded. Socioeconomic variables are also utilized, including education, classified as no formal education (below ISCED 1), primary school (International Standard Classification of Education [ISCED] 1 or 2), secondary school (ISCED 3 or 4), or higher education (ISCED 5 or above). Employment status, categorized as employed, unemployed, or inactive (retirees, students, and those performing domestic tasks, carrying out compulsory service, or unable to work for health reasons) and income level, separated by quintiles within each respective country where the first quintile contains the lowest values and the fifth quintile contains the highest values, comprise the other socioeconomic variables. Also included in the study is a variable utilizing the Oslo Social Support Scale (OSSS-3), a standardized three-question tool for measuring social support as poor support, moderate support, or strong support [20]. Lifestyle variables include smoking (yes/no), vaping (yes/no), alcohol consumption (more than once a month and less than once a month), and physical activity (PA; low, moderate, and high) as defined by the methodology used in Jemna et al. 2022 [21].

This study also contains health and health system variables. To begin with, utilizing clusters created by Oleszkiewicz et al. [12], a variable was created to measure the impact of OTC medication availability on SM. Three clusters were formed using information from the legal acts on the websites of the countries' respective Ministries of Health, as well as from scientific literature. The first cluster, labeled pharmacy-only, contains countries where pharmacies have a monopoly on dispensing medicines: Austria, Belgium, Cyprus, Estonia, Finland, France, Greece, Lithuania, Luxembourg, Latvia, Malta, Slovakia, and Spain. The second cluster, labeled nonpharmacy, refers to countries where individuals may purchase medicines from trading points other than a pharmacy specified in general sales lists: Poland, Ireland, the Netherlands, Slovenia, Hungary, Italy, Czechia, Denmark, and Sweden. The third and final cluster, labeled limited nonpharmacy, encompasses countries with a limited range of medicines (i.e., medicines with a low therapeutic effect such as herbal medicines) sold in limited-service pharmacies (where it is not possible to consult with qualified staff when making a purchase): Bulgaria, Croatia, Germany, Portugal, Romania, and Switzerland. Additional health variables include the presence of a long-standing health problem, use of prescribed medicine in the past 2 weeks, visit to a general practitioner/family doctor, visit to medical or surgical specialist, and visit to a psychologist, psychotherapist, or psychiatrist in the past 12 months, all measured dichotomously (*yes*/*no*). A variable measuring self-perceived health, rated as very good/good, fair, and bad/very bad is also utilized. Finally, five variables evaluating unmet health care needs in the past 12 months with respect to waiting lists, distance or transportation problems, inability to afford medical examination or treatment, inability to afford prescribed medicines, and inability to afford mental health care are included, with possible answers including yes, no, and no need for health care.

### 2.3. Statistical Analysis

We calculated the prevalence of SM in individuals with recognized depression and symptoms of depression according to the PHQ-8 by country and for each independent variable, selected based on relevancy in the literature, via answers to the dependent variable. Furthermore, prevalences are stratified by sex and corresponding female-male odds ratios (ORs) with 95% confidence intervals (95% CIs) provided for all countries and independent variables. To estimate the independent effect of the study variables on SM, we calculated the adjusted ORs (AORs) and the 95%CIs via multivariable logistic regression analysis utilizing the methodology used by Hosmer Jr. et al. [22]. The independent variables for these models were selected using a stepwise variable selection procedure that began with univariate screening (*p*  < 0.25), followed by iterative refinement using Wald tests, partial likelihood ratio tests, and checks for confounding effects. Interaction was evaluated for both sex (male/female) and depression (recognized depression/depression symptoms) to measure the effect of SM. In total, nine multivariable logistic regression models were generated via combinations of the variables related to depression and sex. Estimates were made using the survey command (svy function) in Stata [23], enabling the incorporation of sample design and weights in all statistical calculations. Statistical significance is set as two-tailed *α* < 0.05. The figure was created in R using the ggplot2 package [24, 25].

## 3. Results

**Figure 1:**
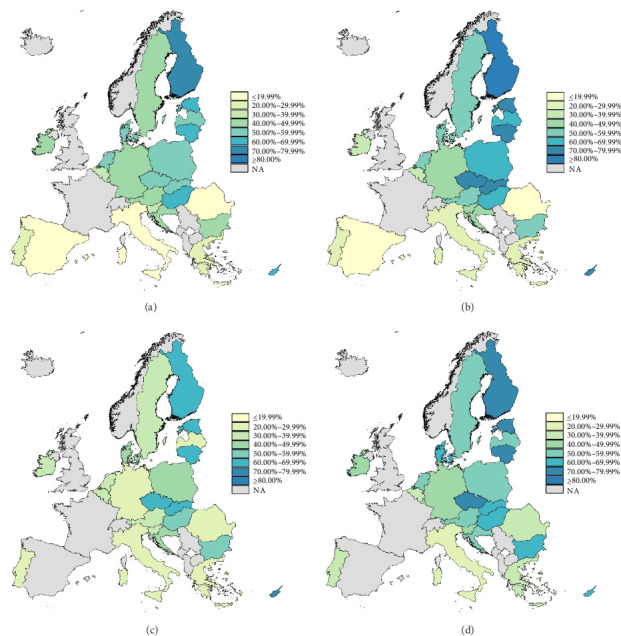
Self-medication prevalence (%) in noninstitutionalized residents aged 15 and over with recognized depression and symptoms of depression in the European Union. European Health Interview Survey Wave 3 (2018–2020). (a) Men with recognized depression. (b) Women with recognized depression. (c) Men with symptoms of depression. (d) Women with symptoms of depression.

The characteristics of the study population are available in Table 1. 33.47% of the sample is male, while the other 66.53% is female. Additionally, 72.13% of the sample pertain to the recognized depression category with the remaining 27.87% belonging to the symptoms of depression group. In Supporting Information 1: Table A, the male and female prevalences for the recognized depression and symptoms of depression groups are stratified by country. These values are also displayed graphically via maps in Figure 1. It can be observed that the lowest and highest prevalence of SM, respectively, is amongst men with recognized depression in Spain at 13.66% (95% CI = 11.14–16.57) and females with recognized depression in Finland at 86.89% (95% CI = 81.64–91.03).

SM prevalence with respect to demographic, socioeconomic, and lifestyle variables is displayed in Table 2. Total SM prevalence in individuals with recognized depression or symptoms of depression in the EU sits at 38.46% (95% CI = 37.54–39.39) in men and 46.84% (95% CI = 46.09–47.58) in women (OR: 1.41; 95% CI = 1.27–1.57). For women, SM prevalence is greater in those meeting PHQ-8 criteria for symptoms of depression than those who have recognized depression (49.87% vs. 45.68%; 95% CI = 48.45–51.30 and 44.80–46.56); in men, the opposite is true (33.25% vs. 40.52%; 95% CI = 31.59–34.95 and 39.42–41.62). In both men and women, SM is most prevalent for those aged 25–44 (44.16% and 56.00%, respectively; 95% CI = 42.42–45.90 and 54.49–57.50). It is also observable that SM prevalence increases with improved education level (no formal education: 15.57% vs. higher education: 59.06%; 95% CI = 12.88–18.61 and 57.29–60.86). Additionally, our findings show that in those with no formal education and recognized depression, men self-medicate more than their female counterparts (OR: 0.53; 95% CI = 0.31–0.86), and that this relationship inverts with greater educational level (higher education OR: 1.72; 95% CI = 1.46–2.01). Meanwhile, Table 3 contains SM prevalence by health and health system variables. The findings establish that SM prevalence in individuals with recognized depression or symptoms of depression is greater for those living in countries with non-pharmacy OTC medication availability versus those who live in countries with pharmacy-only medication sales (male: 42.63% vs. 35.29%, female: 51.91% vs. 40.85%; 95% CIs = male: 41.02–44.24 and 32.91–37.70, female: 50.64–53.17 and 39.12–42.62). Furthermore, the results show that SM prevalence for people with recognized depression or symptoms of depression is lower for those consuming prescription medicine (male: 38.06% vs., 41.32%, female: 46.60% vs. 50.18%; 95% CIs = male: 37.06–39.07 and 38.98–43.75, female: 45.82–47.38 and 47.64–52.69). Additionally, among population with recognized depression or symptoms of depression, we also find that SM prevalence is greater for those who report having unmet health needs in the past 12 months, such as physical difficulties in accessing health care (male: 55.50% vs. 37.88%, female: 52.71% vs. 53.31%; 95% CIs = male: 51.74–59.10 and 33.13–43.09, female: 49.59–55.75 and 48.85–57.70).

The findings of the multivariable analysis of the independent variables are presented in Table 4. In our results, we find that women are more likely to SM (both groups AOR = 1.37; 95% CI = 1.18–1.60). Simultaneously, the findings indicate that men self-medicate less with depression symptoms (AOR = 0.69; 95% CI = 0.56–0.84), while the opposite is true in women (AOR = 1.28; 95% CI = 1.11–1.48). These results also show that in women, age is a significant determinant for SM, with those ages 15–24 (both groups AOR = 1.50; 95% CI = 1.09–2.07) and 25–44 (both groups AOR = 1.47; 95% CI = 1.15–1.89) being most susceptible to self-medicate (AORs calculated vs. ages 75+). Additionally, the findings demonstrate that for the study population, higher education is associated with greater SM when compared with those lacking formal education (AOR = 3.74; 95% CI = 2.83–4.93), especially among women (both groups AOR = 5.63; 95% CI = 4.20–7.54). Furthermore, we find that employment increases in SM in individuals with recognized depression and symptoms of depression (vs. unemployment AOR = 1.45; 95% CI = 1.12–1.88). Moreover, in men with recognized depression, consuming alcohol more than once a month is also associated with greater odds of self-medicating (AOR = 1.36; 95% CI = 1.11–1.66). Our analysis also demonstrates that high levels of PA while depressed is associated with greater SM in contrast with low levels of PA (both groups AOR = 1.38; 95% CI = 1.18–1.60). Finally, we show that individuals with recognized depression self-medicate more in countries with wider accessibility to medication (AOR = 2.10; 95% CI = 1.86–2.36), while the opposite is true for individuals with symptoms of depression (AOR = 0.69; 95% CI = 0.59–0.82).

## 4. Discussion

To the authors' knowledge, this article is the first to detail SM prevalence and associated factors in individuals with recognized depression and symptoms of depression in the EU. Compared to the 34.3% SM prevalence found in the general EU population (28.5% in men and 39.7% in women) [15], SM prevalence among individuals with recognized depression and symptoms of depression is higher at 38.46% (95% CI = 37.54–39.39) and 46.84% (95% CI = 46.09–47.58) for men and women, respectively. Previous studies have linked depression to the NMUP of prescription medicine such as opioids, stimulants, sedatives, and antidepressants in American university students [7], with similar results found in the misuse and NMUP of opioids in a systematic review and a study of Australian patients [5, 6]. OTC analgesic-use for pains directly stemming from depression has also been documented [26]. Furthermore, a Polish survey of doctors found that mental health issues increased the likelihood of a patient self-medicating before visiting a doctor [10]. Our study adds to the observed linkage between medication consumption without prescription and depression on a broader scale than previous studies, including not only medications but also vitamins and herbal medicines. These findings lend support to the SM hypothesis [4], which posits that individuals with mental illness often turn to SM to manage their symptoms. This hypothesis traditionally focuses on the link between depression and the consumption of psychoactive substances [27, 28], but it is noteworthy that SM also includes many self-care practices, such as consuming vitamins or medicinal herbs, that individuals looking to alleviate their depression or its symptoms may adopt. The results also show that SM prevalence in this population varies substantially between EU countries, with the highest cumulative SM prevalence in found in Finland (73.04% in men and 84.80% in women) and the lowest in Italy (19.68% in men and 24.74% in women). This same phenomenon—Northern and Eastern Europe self-medicating more frequently than Western and Southern Europe—is visible in general EU population [15], highlighting the importance of socio-cultural, economic, and governmental factors in shaping our health habits.

One such influence is the availability of OTC medication. It has been logically suggested that increased OTC medication distribution points (such as supermarkets) proliferate SM [12]. The results of this study support this idea, finding that nonpharmacy medication purchasing significantly increases SM (vs. pharmacy-only purchasing AOR = 1.98; 95% CI = 1.78–2.21), though a 2018 Italian study found no statistically significant relationship between alternative distribution points and nonprescription medicine purchasing [29]. In our study, a key difference exists between those with recognized depression and symptoms of depression. Puzzlingly, while those with recognized depression self-medicate more frequently in countries with nonpharmacy sale of medications (vs. pharmacy-only sale: AOR = 2.10; 95% CI = 1.86–2.36), those with symptoms of depression in these countries self-medicate 31% less (vs. pharmacy-only sale: AOR = 0.69; 95% CI = 0.59–0.82). Perhaps participants with symptoms of depression living in countries with more open access to OTC medications see these medications as more commonplace and less useful for dealing with symptoms of depression. On the other hand, those with recognized depression may have identified the value of SM in dealing with their depression and increased access facilitates this usage. Nonetheless, a similarly puzzling result to our study was found in research working with cannabis, where participants living in U.S. states where cannabis is legal were less likely to use it to self-medicate for depression [30], though contradictory results were found in another study [31]. Regardless of the cause, what these results make clear is that further research is required to understand the effect of OTC medication availability on SM, especially given the economic and social benefits of responsible SM in Europe [32].

Additionally, our results reinforce previous research identifying greater SM among women [15]. Specifically, this study supports these findings amongst those with recognized depression (AOR = 1.41; 95% CI = 1.22–1.64) and symptoms of depression (AOR = 2.25; 95% CI = 1.86–2.71). In line with this study, a systematic review by Votaw et al. [33] demonstrated that gender serves as a moderator for the association between symptoms of depression and NMUP of benzodiazepine, with a stronger association found in women compared to men. Interestingly, we also find that men and women exhibit different behaviors with respect to recognized depression and symptoms of depression. While women are more likely to self-medicate with only symptoms of depression rather than recognized depression (AOR = 1.28; 95% CI = 1.11–1.48), men self-medicate less with only symptoms of depression than with recognized depression (AOR = 0.69; 95% CI = 0.56–0.84). This suggests that women may self-medicate proactively in response to depression symptomology, while men may wait until greater recognition of their depression, such as a diagnosis by a medical professional. This is encapsulated by the finding that women with depression symptoms are 75% more likely than men with recognized depression to self-medicate (AOR = 1.75; 95% CI = 1.36–2.26). This result could be explained by previous research showing that women are more proactive health decision makers, including seeking for external health information and displaying concern about health consequences [34]. Simultaneously, women are often conditioned to turn to medication [35], while men are accustomed to “toughing it out” and avoiding medication [36].

Along the same lines, multiple other variables display differing relationships for men and women, highlighting differences in how they self-medicate and deal with depression. First, we find that age is an influential variable specifically for women, with those aged 15–24 (symptoms of depression AOR = 1.60; 95% CI = 1.02–2.51) and 25–44 (symptoms of depression AOR = 1.93; 95% CI = 1.33–2.78) most likely to self-medicate. Previous research has found an age discrepancy between the peaks in prevalence of depression (ages 14–25) and antidepressant use (ages 45+) [37], which, coupled with our findings, suggests that young women may SM their depression until later receiving antidepressant treatment, especially for those with only symptoms of depression. Providing further backing for this idea, a study from the United States found that onset of depressive disorder at earlier ages was associated with higher likelihood of NMUP [38]. Second, this study finds that while education is influential for men at the highest level of education (both groups vs. no formal education AOR = 1.67; 95% CI = 1.02–2.71), each level of improved education increases the probability of SM in women, particularly for women symptoms of depression (higher education vs. no formal education AOR = 3.37; 95% CI = 2.04–5.57). Education has been strongly correlated with SM across a multitude of demographics, including general population in the EU [15]. Improved health literacy, greater confidence to make self-care decisions, increased questioning of doctor's orders, and improved social networking (which leads to medication sharing) are just a few of the reasons why education is positively associated with SM [15]. The results of this study suggest these motivations are amplified for women with symptoms of depression and less present for other study groups.

Third, we find that being employed (vs. unemployed) is significantly associated with SM (both groups and sexes AOR = 1.45; 95% CI = 1.12–1.88), perhaps due to time limitations for doctor's visits for the employed. Interestingly, this relationship for men is found in those with symptoms of depression, while in women, it is for those with recognized depression, which may reflect distinct motivations for why men and women self-medicate. For example, employed men with symptoms of depression may self-medicate in response to stresses from work, while employed men with recognized depression may already have other methods for dealing with job stress. Fourth, we find a noteworthy relationship between SM and alcohol in men. Specifically, SM is significantly associated with alcohol consumption in men with recognized depression (AOR = 1.42; 95% CI = 1.12–1.81), corroborating previous studies linking alcohol consumption to SM [15, 39]. Given the vulnerability of this population and incompatibility between antidepressants and alcohol, this potentially dangerous phenomenon should be further investigated.

Fifth, our findings unveil that the use of prescribed medicine for men with depressive symptoms is significantly associated with SM (AOR = 1.68; 95% CI = 1.12–2.52). The same was not found in general EU population as there is typically a trade-off between prescription and non-prescription medicines [15], suggesting that this may be a trait unique to men with depressive symptoms. Contrarily, in all female study groups but no male study groups, SM is associated with visits to medical or surgical specialists (both groups AOR = 1.32; 95% CI = 1.13–1.53), whereas in EU general population, this relationship was found in both men and women. This may be suggestive of reduced self-care by men with recognized depression or symptoms of depression compared to general male population in the EU. Surprisingly, visits to mental health specialists nor general practitioners (who often serve as an entry point for treatment of patients with depression) [40] did not factor into our multivariable models.

Nonetheless, men and women with recognized depression and symptoms of depression are similar in terms of SM with respect to several other variables. Our study finds that engaging in high levels of PA is associated with SM (vs. low PA, total both sexes AOR = 1.38; 95% CI = 1.18–1.60). This is likely explained by overlapping self-care behaviors between PA and concern about one's health, which may include self-medicating with vitamins and other nutritional supplements. Our results also suggest that SM is motivated by difficulties in accessing health care systems due to distance or transportation problems for both men (both groups AOR = 1.89; 95% CI = 1.18–3.04) and women (both groups AOR = 1.55; 95% CI = 1.20–1.99). This highlights the importance of increasing healthcare accessibility, especially in rural or other poorly communicated areas. Interestingly, inability to afford mental health care did not contribute to our multivariable models, though lack of ability to afford general treatment/examination did in the model for both sexes/groups (AOR = 1.38; 95% CI = 1.07–1.79). Previous research found similar relationships between SM and healthcare access, as well as SM and exercise, in general EU population [15], suggesting these variables behave similarly in this demographic and in the general population.

Finally, this study is subject to a series of limitations. First, due to the cross-sectional nature of the data, causality cannot be determined. Second, the European Health Survey does not identify specific active pharmaceutical ingredients nor does it identify groups of medicines for specific diseases, conditions, or disorders. It is also worth noting that the EHIS references sex, which may inadequately capture the range of diversity and social, political, and economic forces expressed in gender [41]. Additionally, it is possible that social desirability bias could have led to underreporting on SM, depression, and symptoms of depression, affecting both prevalences and associated factors. This is furthered by the fact that differences existed between countries in data collection methods and sampling design, potentially altering results and reducing comparability between countries [42]. For this study, individuals interviewed face-to-face or over the phone may have felt additional social pressure to underreport SM, depression, and symptoms of depression vs. those who filled out self-administered written or online surveys.

Moreover, EHIS data collection occurred nonsimultaneously across the EU over multiple years and seasons, and consequently, seasonal and annual variance of conditions that provoke SM and depression could have been impactful. Specifically, lower temperatures are associated with greater OTC respiratory medication sales [43], and as a result, SM prevalence could be greater in colder months. Simultaneously, depression is also more frequent in colder months, which could alter consumption habits and the strength of the relationship [44]. The vast majority of EHIS responses were collected in autumn. Along the same lines, three countries (Germany, Spain, and Malta) collected data after the beginning of the COVID-19 pandemic, which could alter recognized depression, symptoms of depression, and SM prevalences [45]. An additional limitation is that the data is self-reported and thus susceptible to recall bias, though the narrow 2-week window of the dependent variable question and PHQ-8 questions should have reduced some of this effect. Additionally, the nonresponse rate, ranging from 12% to 78% across countries [45], may be influential as those who did not participate could have shared insights into SM, even if the direction of this effect is indeterminable. Similarly, Spain's nonparticipation in the PHQ questions used to form the symptoms group could have also impacted the findings. Finally, criticism has been levied against the utility of the PHQ-8 [46]; however, these criticisms are oriented principally toward its use as a measure of depression prevalence, not the presence of symptoms of depression as used in this study. Studies have shown that compared to the PHQ-9 (which includes an additional question about suicide), the PHQ-8 undergoes minimal sensitivity loss and no specificity loss [47].

Nonetheless, the quality report of the third wave of the EHIS details that the data underwent validation, calibration, and nonresponse adjustments procedures to minimize the effect of all potential sources of sampling and nonsampling errors, resulting in dataset that is highly harmonized and allows for a high degree of comparability across EU member states [45]. Coupled with the robust weighted sample size of 25,701, the authors feel that none of the aforementioned limitations should dampen the strength or relevancy of the findings.

## 5. Conclusion

In conclusion, this study finds that individuals with recognized depression or depressive symptoms engage in SM more frequently than the general population, and there are differences in the factors affecting their consumption. A key finding of this study is that the availability of OTC medications outside of pharmacies increases their usage among individuals with recognized depression or depressive symptoms. Furthermore, this study reveals differences in SM between men and women with recognized depression and symptoms of depression. Specifically, women were more likely to self-medicate in response to depressive symptoms, while men did so more often when depression was formally recognized. Other influencing variables such as age, education, employment, alcohol consumption, prescription drug use, and healthcare utilization also showed distinct associations in men and women. In contrast, factors like physical activity and difficulty accessing healthcare behave similarly in men and women. Going forward, research should take a closer look into SM by vulnerable populations, such as individuals suffering from depression, to ensure their consumption is informed, safe, and beneficial. Additionally, given the variability in medication access across member states and its significant impact on consumption, there is a need for greater examination of regulations surrounding OTC medication access.

## Figures and Tables

**Table 1:** Descriptive table of depressed and noninstitutionalized residents aged 15 and over in the European Union.

Independent variables	Male (*N* = 8602 [33.47%])		Female (*N* = 17,099 [66.53%])		Both sexes (*N* = 25,701 [100%])	
	*N*	%	*N*	%	*N*	%
Age (years)						
15–24	618	9.20	1117	8.76	1735	8.93
25–44	1903	29.41	2996	24.38	4899	26.31
45–64	3066	38.15	5868	34.61	8934	35.97
65–74	1417	10.99	2939	13.13	4356	12.31
75+	1598	12.24	4179	19.13	5777	16.49
Nationality*⁣*^*∗*^						
Native-born	7520	86.07	15,155	88.16	22,675	87.36
Born in another EU state	323	3.23	718	4.16	1041	3.81
Born in non-EU country	739	10.70	1198	7.68	1937	8.83
Degree of urbanization						
Cities	3227	44.00	6335	44.02	9562	44.01
Towns and suburbs	2682	33.35	5431	33.08	8113	33.18
Rural areas	2663	22.65	5271	22.90	7934	22.81
Marital status						
Single	2516	38.77	2986	22.38	5502	28.67
Married or living with partner	4456	41.94	7578	44.42	12,034	43.47
Divorced	699	7.21	4353	20.27	5052	15.26
Widowed	913	12.07	2122	12.92	3035	12.60
Education level						
No formal education	262	1.88	929	3.63	1191	2.96
Primary school	2863	30.03	6638	36.96	9501	34.30
Secondary school	3400	50.42	5740	42.24	9140	45.37
Higher education	1963	17.67	3597	17.18	5560	17.37
Employment status						
Employed	2720	39.10	4720	33.18	7440	35.44
Unemployed	714	9.63	1007	7.36	1721	8.23
Inactive	5071	51.28	11,227	59.46	16,298	56.33
Income level						
1st quintile	2067	29.57	4695	29.98	6762	29.82
2nd quintile	1837	22.78	4121	24.42	5958	23.79
3rd quintile	1703	19.62	3011	18.79	4714	19.11
4th quintile	1372	15.79	2462	15.23	3834	15.45
5th quintile	1138	12.24	1859	11.58	2997	11.83
Oslo Social Support Scale						
Poor support	5766	63.35	12,115	69.16	17,881	66.92
Moderate support	2377	34.70	4192	29.00	6569	31.19
Strong support	112	1.95	182	1.85	294	1.89
Smoking						
Yes	2660	40.26	3592	26.88	6252	32.01
No	5844	59.74	13,278	73.12	19,122	67.99
Vaping						
Yes	364	6.52	469	3.83	833	4.86
No	8078	93.48	16,306	96.17	24,384	95.14
Alcohol consumption						
More than once a month	2931	40.20	2949	20.62	5880	28.17
Once a month or less	4636	59.80	12,083	79.38	16,719	71.83
Physical activity						
Low physical activity	5325	55.73	11,437	60.61	16,762	58.74
Moderate physical activity	1343	15.09	2633	16.49	3976	15.95
High physical activity	1875	29.19	2905	22.90	4780	25.31
Medication availability cluster						
Pharmacy-only	2285	14.32	5022	17.64	7307	16.36
Nonpharmacy	3854	33.79	6956	34.94	10,810	34.50
Limited pharmacy	2463	51.90	5121	47.42	7584	49.13
Long-standing health problem						
Yes	6982	78.29	14,191	82.13	21,173	80.66
No	1564	21.71	2784	17.87	4348	19.34
Self-perceived health						
Very good/good	2237	30.88	4178	28.50	6415	29.41
Fair (neither good nor bad)	3047	34.66	6872	40.72	9919	38.40
Bad/very bad	3281	34.45	5966	30.78	9247	32.19
Use of prescribed medicine in the past 2 weeks						
Yes	7275	84.68	15,377	91.18	22,652	88.68
No	1288	15.32	1661	8.82	2949	11.32
Visit to a general practitioner or family doctor*⁣*^*∗∗*^						
Yes	6508	71.72	14,042	80.19	20,550	76.94
No	2053	28.28	2998	19.81	5051	23.06
Visit to a medical or surgical specialist*⁣*^*∗∗*^						
Yes	5553	64.02	11,924	72.80	17,477	69.44
No	2964	35.98	5026	27.20	7990	30.56
Visit to a psychologist, psychotherapist, or psychiatrist*⁣*^*∗∗*^						
Yes	2469	33.92	5161	37.28	7630	36.00
No	6012	66.08	11,750	62.72	17,762	64.00
Unmet need for health care due to waiting lists*⁣*^*∗∗*^						
Yes	2207	26.57	5080	30.88	7287	29.23
No	5142	59.51	10,089	58.96	15,231	59.17
No need for health care	1174	13.91	1799	10.15	2973	11.59
Unmet need for health care due to distance or transportation problems*⁣*^*∗∗*^						
Yes	736	9.99	1535	8.75	2271	9.23
No	6618	77.49	13,510	80.62	20,128	79.42
No need for health care	1165	12.52	1885	10.63	3050	11.35
Unmet need for health care due to inability to afford medical examination or treatment*⁣*^*∗∗*^						
Yes	1003	10.17	2333	13.10	3336	11.97
No	5794	72.03	11,696	72.31	17,490	72.20
No need for health care	1306	17.80	2171	14.59	3477	15.82
Unmet need for health care due to inability to afford prescribed medicines*⁣*^*∗∗*^						
Yes	849	9.61	1989	11.61	2838	10.84
No	6201	76.42	12,673	78.22	18,874	77.53
No need for health care	1047	13.97	1524	10.17	2571	11.63
Unmet need for health care due to inability to afford mental health care*⁣*^*∗∗*^						
Yes	699	9.53	1566	9.80	2265	9.70
No	4548	61.54	8845	61.38	13,393	61.44
No need for health care	2847	28.93	5773	28.81	8620	28.86
Self-medication in the past 2 weeks						
Yes	3282	38.46	8013	46.84	11,295	43.63
No	5320	61.54	9086	53.16	14,406	56.37
Depression						
Recognized	5752	71.66	11,941	72.41	17,693	72.13
Symptoms	2850	28.34	5158	27.59	8008	27.87

*Note:* European Health Interview Survey Wave 3 (2018–2020). *⁣*^*∗*^ = does not include data from Malta; *⁣*^*∗∗*^ = in the past 12 months. Inactive = retirees, students, and those performing domestic tasks, carrying out compulsory service, or unable to work for health reasons. 1st quintile contains the lowest values and the 5th quintile the highest values.

Abbreviations: EU, European Union; OR, odds ratio.

**Table 2:** Self-medication prevalence by sex in depressed and noninstitutionalized residents aged 15 and over in the European Union according to demographic, socioeconomic, and lifestyle variables.

Independent variables	Recognized depression			Depression symptoms*⁣*^*∗*^			Both groups		
	Male	Female	OR female	Male	Female	OR female	Male	Female	OR female
Total	40.52 (39.42–41.62)	45.68 (44.80–46.56)	1.23 (1.08–1.41)	33.25 (31.59–34.95)	49.87 (48.45–51.30)	1.99 (1.66–2.39)	38.46 (37.54–39.39)	46.84 (46.09–47.58)	1.41 (1.27–1.57)
Age^a,b,c,d,e,f^									
15–24 years	47.73 (43.92–51.41)	49.12 (46.11–52.23)	1.06 (0.64–1.73)	25.19 (20.66–30.48)	51.67 (47.20–56.12)	3.17 (2.53–4.08)	40.84 (37.79–43.93)	49.93 (47.37–52.43)	1.44 (0.97–2.14)
25–44 years	44.35 (42.38–46.35)	54.48 (52.74–56.20)	1.50 (1.14–1.97)	43.53 (39.95–47.09)	60.76 (57.74–63.74)	2.00 (1.28–3.15)	44.16 (42.42–45.90)	56.00 (54.49–57.50)	1.60 (1.27–2.03)
45–64 years	38.12 (36.43–39.86)	44.67 (43.24–46.11)	1.31 (1.07–1.60)	28.80 (26.05–31.68)	53.57 (50.90–56.27)	2.85 (2.07–3.92)	35.84 (34.39–37.34)	46.65 (45.39–47.93)	1.56 (1.31–1.86)
65–74 years	35.81 (32.48–39.26)	41.38 (39.06–43.72)	1.26 (0.92–1.74)	37.99 (33.29–42.67)	45.42 (41.18–49.58)	1.36 (0.91–2.02)	36.57 (33.85–39.36)	42.34 (40.30–44.37)	1.27 (0.99–1.63)
75+ years	36.20 (32.79–39.79)	35.50 (33.38–37.68)	0.97 (0.69–1.35)	28.67 (25.21–32.54)	39.46 (36.88–42.03)	1.62 (1.22–2.15)	32.84 (30.32–35.40)	37.17 (35.53–38.84)	1.21 (0.96–1.52)
Nationality*⁣*^*∗*^^a,b,c,f^									
Native-born	39.40 (38.21–40.58)	45.20 (44.26–46.14)	1.27 (1.10–1.45)	34.16 (32.34–36.00)	49.83 (48.32–51.33)	1.91 (1.58–2.32)	37.92 (36.93–38.92)	46.50 (45.70–47.29)	1.42 (1.27–1.59)
Born in another EU state	41.32 (34.88–47.65)	55.62 (51.32–59.77)	1.78 (0.85–3.71)	30.14 (22.18–38.63)	49.53 (42.29–56.64)	2.27 (1.68–3.22)	37.51 (32.60–42.82)	54.05 (50.33–57.63)	1.96 (1.05–3.64)
Born in non-EU country	48.17 (44.75–51.59)	44.71 (41.65–47.78)	0.87 (0.54–1.39)	24.28 (19.92–29.30)	50.44 (44.90–56.08)	3.17 (2.54–4.07)	41.44 (38.62–44.34)	46.04 (43.33–48.71)	1.20 (0.82–1.77)
Degree of urbanization^a,c,d,e,f^									
Cities	41.98 (40.32–43.65)	46.19 (44.87–47.52)	1.19 (0.97–1.44)	36.68 (34.01–39.36)	52.29 (50.09–54.47)	1.89 (1.40–2.54)	40.55 (39.13–41.97)	47.83 (46.69–48.96)	1.34 (1.14–1.58)
Towns and suburbs	39.40 (37.54–41.34)	44.09 (42.57–45.61)	1.21 (0.96–1.53)	30.84 (28.00–33.87)	48.76 (46.28–51.33)	2.13 (1.49–3.05)	37.08 (35.47–38.68)	45.35 (44.05–46.66)	1.40 (1.15–1.71)
Rural areas	38.46 (36.09–40.89)	46.53 (44.65–48.40)	1.39 (1.24–1.57)	30.82 (27.68–34.09)	47.33 (44.46–50.20)	2.01 (1.55–2.62)	35.90 (33.99–37.85)	46.77 (45.21–48.35)	1.57 (1.29–1.91)
Marital status^a,b,c,d,e,f^									
Single	43.23 (41.53–44.95)	49.98 (48.13–51.80)	1.31 (1.00–1.71)	35.06 (32.04–38.23)	57.07 (53.91–60.13)	2.46 (1.58–3.83)	41.43 (39.95–42.95)	51.78 (50.20–53.37)	1.52 (1.21–1.90)
Married or living with partner	38.70 (37.00–40.44)	46.04 (44.76–47.34)	1.35 (1.15–1.59)	35.76 (33.31–38.32)	50.34 (48.06–52.58)	1.82 (1.46–2.27)	37.78 (36.36–39.20)	47.10 (45.98–48.22)	1.47 (1.28–1.67)
Divorced	34.68 (30.19–39.18)	38.30 (36.24–40.37)	1.17 (0.60–2.04)	27.24 (22.75–32.18)	43.47 (40.86–46.16)	2.05 (1.30–3.25)	31.38 (28.10–34.64)	40.30 (38.68–41.94)	1.48 (1.01–2.16)
Widowed	39.85 (36.66–42.97)	46.56 (44.19–48.93)	1.31 (0.90–1.91)	24.56 (20.28–29.04)	53.09 (48.79–57.40)	3.47 (1.95–6.18)	35.48 (32.90–38.12)	48.08 (46.02–50.18)	1.68 (1.22–2.32)
Education level^a,b,c,d,e,f^									
No formal education	25.44 (18.44–32.98)	13.10 (10.33–16.36)	0.44 (0.18–0.82)	26.87 (16.95–38.25)	24.29 (17.65–31.96)	0.87 (0.51–1.49)	25.90 (20.05–32.17)	15.57 (12.88–18.61)	0.53 (0.31–0.86)
Primary school	32.50 (30.54–34.48)	36.10 (34.69–37.53)	1.17 (0.88–1.56)	24.13 (21.54–26.85)	41.33 (39.14–43.57)	2.21 (1.57–3.12)	29.87 (28.30–31.48)	37.68 (36.49–38.89)	1.42 (1.13–1.78)
Secondary school	43.61 (42.04–45.16)	51.65 (50.31–52.99)	1.38 (1.13–1.68)	35.20 (32.75–37.68)	55.86 (53.59–58.11)	2.32 (1.73–3.13)	41.34 (40.03–42.68)	52.74 (51.58–53.89)	1.58 (1.34–1.87)
Higher education	45.37 (42.75–48.03)	58.00 (55.92–60.10)	1.66 (1.37–2.01)	46.48 (42.06–50.78)	61.96 (58.55–65.36)	1.87 (1.40–2.51)	45.67 (43.40–47.92)	59.06 (57.29–60.86)	1.72 (1.46–2.01)
Employment status^a,b,c,d,e,f^									
Employed	38.96 (36.91–41.02)	44.27 (42.67–45.91)	1.48 (1.19–1.82)	24.47 (21.70–27.43)	48.76 (46.04–51.49)	1.83 (1.29–2.59)	34.82 (33.14–36.54)	45.45 (44.07–46.86)	1.56 (1.30–1.87)
Unemployed	43.58 (41.19–45.96)	41.88 (40.11–43.70)	1.09 (0.67–1.78)	29.95 (26.56–33.49)	45.14 (42.25–48.13)	4.80 (2.23–10.37)	39.66 (37.67–41.65)	42.78 (41.25–44.31)	1.42 (0.92–2.20)
Inactive	40.13 (37.62–42.70)	44.84 (42.78–46.90)	1.13 (095–1.35)	36.49 (32.60–40.45)	51.84 (48.47–55.23)	2.11 (1.72–2.60)	39.09 (36.96–41.23)	46.74 (45.00–48.53)	1.37 (1.19–1.57)
Income level^a,b,c,d,e,f^									
1st quintile	38.17 (35.44–41.01)	49.04 (46.76–51.33)	1.24 (0.94–1.65)	41.87 (37.50–46.65)	52.61 (48.76–56.35)	2.94 (1.95–4.42)	39.19 (36.81–41.57)	49.99 (48.04–51.96)	1.56 (1.23–1.97)
2nd quintile	43.04 (39.93–46.32)	49.63 (46.98–52.35)	0.93 (0.69–1.26)	42.52 (37.24–47.91)	56.41 (52.28–60.46)	1.92 (1.37–2.71)	42.90 (40.22–45.71)	51.65 (49.39–53.88)	1.14 (0.89–1.45)
3rd quintile	43.23 (41.48–44.98)	53.03 (51.51–54.51)	1.21 (0.91–1.61)	44.43 (41.43–47.42)	59.37 (56.77–61.91)	1.87 (1.51–3.04)	43.54 (42.05–45.07)	54.60 (53.30–55.89)	1.37 (1.07–1.75)
4th quintile	35.81 (32.58–39.26)	37.94 (35.06–40.88)	1.56 (1.18–2.05)	20.16 (15.43–25.85)	54.83 (47.65–61.78)	1.54 (1.06–2.24)	32.32 (29.46–35.21)	40.48 (37.77–43.20)	1.55 (1.24–1.94)
5th quintile	39.23 (37.67–40.82)	42.24 (41.08–43.39)	1.30 (0.96–1.77)	28.10 (26.03–30.28)	45.29 (43.55–47.06)	1.75 (1.12–2.72)	35.70 (34.43–36.98)	43.17 (42.21–44.14)	1.42 (1.10–1.83)
Oslo Social Support Scale^a,b,c,e^									
Poor support	38.44 (37.03–39.86)	44.13 (43.06–45.20)	1.26 (1.08–1.47)	33.05 (31.00–35.20)	49.46 (47.68–51.26)	1.98 (1.58–2.49)	36.83 (35.67–38.01)	45.54 (44.63–46.46)	1.43 (1.26–1.63)
Moderate support	44.12 (42.22–46.02)	49.78 (48.08–51.47)	1.25 (0.97–1.62)	32.33 (29.36–35.39)	50.17 (47.59–52.84)	2.10 (1.52–2.92)	41.05 (39.44–42.67)	49.89 (48.46–51.31)	1.43 (1.16–1.76)
Strong support	24.74 (18.47–32.52)	46.93 (39.96–53.67)	2.69 (0.73–9.90)	48.58 (36.21–62.06)	42.54 (33.25–52.31)	0.78 (0.23–1.91)	31.38 (25.50–38.35)	45.46 (39.82–51.00)	1.82 (0.68–4.87)
Smoking^a,e^									
Yes	43.96 (42.24–45.69)	46.07 (44.44–47.70)	1.09 (0.84–1.40)	33.36 (30.59–36.16)	52.57 (49.46–55.66)	2.21 (1.43–3.41)	41.24 (39.76–42.71)	47.48 (46.03–48.92)	1.29 (1.04–1.60)
No	38.18 (36.76–39.62)	45.73 (44.69–46.78)	1.36 (1.18–1.57)	33.24 (31.16–35.39)	49.44 (47.82–51.04)	1.96 (1.63–2.35)	36.69 (35.52–37.89)	46.83 (45.95–47.71)	1.52 (1.35–1.70)
Vaping^b,c,d,e,f^									
Yes	44.15 (39.72–48.67)	58.96 (54.66–63.14)	1.82 (0.96–3.44)	41.46 (35.07–48.11)	59.54 (50.85–67.28)	2.07 (1.36–3.43)	43.30 (39.65–47.04)	59.08 (55.20–62.75)	1.89 (1.08–3.31)
No	40.41 (39.28–41.56)	45.28 (44.38–46.18)	1.22 (1.07–1.39)	32.77 (31.05–34.55)	50.01 (48.54–51.46)	2.05 (1.71–2.45)	38.27 (37.31–39.23)	46.60 (45.83–47.36)	1.41 (1.26–1.57)
Alcohol consumption^a,b,c,e,f^									
More than once a month	46.88 (45.07–48.69)	49.43 (47.46–51.40)	1.11 (0.87–1.41)	36.77 (33.95–39.70)	53.74 (50.24–57.16)	1.99 (1.39–2.87)	44.15 (42.62–45.70)	50.48 (48.78–52.20)	1.29 (1.05–1.57)
Once a month or less	36.75 (35.31–38.21)	45.08 (44.05–46.10)	1.41 (1.19–1.67)	31.28 (29.12–33.53)	50.91 (49.24–52.55)	2.28 (1.80–2.88)	35.19 (33.98–36.41)	46.71 (45.84–47.58)	1.61 (1.40–1.86)
Physical activity^a,b,c,d,e,f^									
Low physical activity	36.51 (35.04–37.99)	41.20 (40.08–42.33)	1.22 (1.02–1.45)	29.42 (27.38–31.56)	46.64 (44.87–48.42)	2.09 (1.72–2.55)	34.32 (33.12–35.54)	42.79 (41.84–43.75)	1.43 (1.25–1.64)
Moderate physical activity	43.09 (40.24–45.96)	48.81 (46.62–50.97)	1.26 (0.94–1.68)	39.16 (34.64–43.57)	49.79 (46.4–53.35)	1.54 (0.91–2.62)	41.98 (39.56–44.39)	49.08 (47.25–50.94)	1.33 (1.03–1.72)
High physical activity	46.44 (44.43–48.43)	54.87 (53.10–56.64)	1.40 (1.06–1.84)	38.83 (35.35–42.43)	61.73 (58.5–64.88)	2.54 (1.59–4.05)	44.66 (42.91–46.41)	56.43 (54.88–57.98)	1.60 (1.27–2.03)

*Note:* 1st quintile contains the lowest values and the 5th quintile the highest values. European Health Interview Survey Wave 3 (2018–2020). Inactive, retirees, students, and those performing domestic tasks, carrying out compulsory service, or unable to work for health reasons. *⁣*^*∗*^ = according to the PHQ-8; *⁣*^*∗∗*^ = does not include data from Malta.

Abbreviations: EU, European Union; OR, odds ratio.

^a^Statistically significant self-medication differences in men with recognized depression.

^b^Statistically significant self-medication differences in women with recognized depressed.

^c^Statistically significant self-medication differences in men with depression symptoms.

^d^Statistically significant self-medication differences in women with depression symptoms.

^e^Statistically significant self-medication differences in men for both groups.

^f^Statistically significant self-medication differences in women for both groups.

**Table 3:** Self-medication prevalence by sex in depressed and noninstitutionalized residents aged 15 and over in the European Union according to health variables.

Independent variables	Recognized depression			Depression symptoms*⁣*^*∗*^			Both groups		
	Male	Female	OR female	Male	Female	OR female	Male	Female	OR female
Medication availability cluster^a,b,c,d,e,f^									
Pharmacy-only	32.66 (30.09–35.27)	37.45 (35.58–39.30)	1.23 (1.04–1.46)	47.41 (41.39–53.18)	61.40 (56.73–65.91)	1.76 (1.34–2.30)	35.29 (32.91–37.70)	40.85 (39.12–42.62)	1.26 (1.09–1.47)
Nonpharmacy	45.42 (43.42–47.43)	52.28 (50.68–53.85)	1.32 (1.16–1.49)	37.30 (34.63–40.01)	51.28 (49.19–53.38)	1.77 (1.49–2.09)	42.63 (41.02–44.24)	51.91 (50.64–53.17)	1.45 (1.31–1.61)
Limited nonpharmacy	40.09 (38.58–41.61)	45.06 (43.81–46.32)	1.22 (0.96–1.55)	27.39 (25.16–29.65)	46.09 (43.98–48.22)	2.26 (1.57–3.25)	36.62 (35.37–37.91)	45.33 (44.25–46.41)	1.43 (1.18–1.74)
Long-standing health problem^a,b,e^									
Yes	38.78 (37.55–40.01)	46.21 (45.26–47.16)	1.36 (1.17–1.56)	34.01 (32.06–35.98)	49.51 (47.85–51.16)	1.90 (1.53–2.35)	37.49 (36.45–38.53)	47.04 (46.21–47.86)	1.48 (1.31–1.67)
No	47.27 (44.81–49.78)	43.23 (40.97–45.47)	0.85 (0.61–1.18)	30.99 (27.82–34.41)	50.77 (47.96–53.63)	2.29 (1.60–3.29)	41.93 (39.91–43.94)	46.18 (44.43–47.97)	1.19 (0.92–1.53)
Self-perceived health^a,b,d,e,f^									
Very good/good	43.66 (41.70–45.63)	48.25 (46.63–49.89)	1.20 (0.94–1.53)	34.18 (30.98–37.37)	50.57 (47.79–53.32)	1.97 (1.35–2.86)	41.23 (39.54–42.9)	48.85 (47.45–50.25)	1.36 (1.11–1.66)
Fair	37.64 (35.87–39.49)	46.85 (45.51–48.20)	1.46 (1.19–1.79)	35.32 (32.35–38.44)	51.63 (49.25–54.02)	1.95 (1.38–2.76)	37.05 (35.50–38.61)	48.01 (46.84–49.18)	1.57 (1.32–1.87)
Bad/very bad	40.70 (38.78–42.67)	41.45 (39.80–43.08)	1.03 (0.81–1.31)	30.84 (28.30–33.46)	47.51 (45.23–49.86)	2.03 (1.59–2.59)	37.40 (35.85–38.98)	43.51 (42.17–44.85)	1.29 (1.07–1.55)
Use of prescribed medicine^a,b,e,f^									
Yes	39.80 (38.61–41.00)	45.46 (44.55–46.38)	1.26 (1.10–1.45)	33.64 (31.82–35.49)	49.65 (48.15–51.16)	1.94 (1.59–2.38)	38.06 (37.06–39.07)	46.60 (45.82–47.38)	1.42 (1.27–1.59)
No	45.41 (42.52–48.25)	49.08 (46.07–52.18)	1.16 (0.79–1.70)	31.33 (27.23–35.57)	52.53 (47.92–56.83)	2.42 (1.61–3.65)	41.32 (38.98–43.75)	50.18 (47.64–52.69)	1.40 (1.06–1.93)
Visit to a general practitioner or family doctor*⁣*^*∗∗*^^a,c,e^									
Yes	37.85 (36.58–39.12)	45.58 (44.62–46.56)	1.37 (1.19–1.59)	34.86 (32.82–36.96)	49.20 (47.55–50.83)	1.81 (1.46–2.24)	37.06 (35.97–38.14)	46.52 (45.69–47.36)	1.47 (1.31–1.66)
No	48.23 (46.08–50.43)	46.33 (44.27–48.39)	0.93 (0.68–1.25)	30.09 (27.29–33.03)	52.16 (49.28–55.05)	2.53 (1.80–3.57)	42.32 (40.58–44.11)	48.31 (46.63–49.99)	1.27 (1.00–1.61)
Visit to a medical or surgical specialist*⁣*^*∗∗*^^b,d,f^									
Yes	40.73 (39.37–42.12)	46.99 (45.97–48.01)	1.29 (1.10–1.51)	34.23 (32.14–36.42)	54.79 (53.03–56.50)	2.32 (1.85–2.92)	38.92 (37.77–40.09)	48.98 (48.09–49.85)	1.51 (1.32–1.71)
No	40.51 (38.65–42.36)	41.98 (40.27–43.73)	1.06 (0.84–1.35)	31.73 (29.08–34.53)	39.89 (37.43–42.35)	1.43 (1.05–1.93)	37.93 (36.39–39.47)	41.30 (39.89–42.72)	1.15 (0.95–1.39)
Visit to a psychologist, psychotherapist, or psychiatrist*⁣*^*∗∗*^^b,d,e,f^									
Yes	40.37 (38.68–42.06)	50.20 (48.89–51.50)	1.49 (1.22–1.82)	35.86 (31.09–40.82)	57.64 (54.11–61.13)	2.43 (2.01–2.99)	39.90 (38.32–41.52)	51.08 (49.86–52.31)	1.57 (1.30–1.89)
No	40.65 (39.20–42.11)	42.00 (40.82–43.17)	1.06 (0.88–1.26)	32.95 (31.16–34.75)	48.37 (46.82–49.94)	1.91 (1.57–2.32)	37.76 (36.62–38.89)	44.34 (43.40–45.29)	1.31 (1.15–1.50)
Unmet need for health care due to waiting lists*⁣*^*∗∗*^^a,b,c,d,e,f^									
Yes	44.75 (42.64–46.86)	51.60 (50.01–53.16)	1.31 (1.02−1.69)	39.64 (36.07–43.37)	56.13 (53.49–58.68)	1.95 (1.31–2.88)	43.51 (41.68–45.34)	52.80 (51.47–54.16)	1.45 (1.17–1.79)
No	37.33 (35.93–38.77)	42.52 (41.40–43.66)	1.24 (1.05–1.47)	31.63 (29.53–33.75)	47.85 (46.00–49.74)	1.98 (1.60–2.46)	35.66 (34.48–36.85)	43.97 (43.01–44.95)	1.41 (1.23 (1.62)
No need for health care	46.23 (43.16–49.29)	47.14 (44.33–50.04)	1.03 (0.82–1.25)	30.72 (26.63–35.05)	44.99 (40.96–49.16)	1.84 (1.06–3.21)	41.41 (38.93–43.95)	46.44 (44.14–48.83)	1.23 (0.90–1.67)
Unmet need for health care due to distance or transportation problems*⁣*^*∗∗*^^a,b,e,f^									
Yes	55.50 (51.74–59.10)	52.71 (49.59–55.75)	0.89 (0.53–1.50)	37.88 (33.13–43.09)	53.31 (48.85–57.70)	1.87 (1.23–2.21)	49.47 (46.48–52.49)	52.90 (50.34–55.40)	1.15 (0.75–1.74)
No	38.44 (37.21–39.68)	44.70 (43.74–45.67)	1.29 (1.12–1.49)	33.32 (31.39–35.29)	49.73 (48.11–51.37)	1.98 (1.62–2.41)	37.05 (36.01–38.09)	46.02 (45.19–46.86)	1.45 (1.28–1.63)
No need for health care	43.22 (40.08–46.45)	48.93 (46.11–51.74)	1.25 (1.02–1.59)	29.99 (25.64–34.55)	48.28 (44.37–52.33)	2.18 (1.36–3.48)	39.20 (36.60–41.85)	48.71 (46.44–51.03)	1.47 (1.11–1.96)
Unmet need for health care due to inability to afford medical examination or treatment*⁣*^*∗∗*^^a,b,c,d,e,f^									
Yes	50.41 (46.69–54.01)	52.14 (49.57–54.64)	1.07 (0.71–1.61)	37.38 (32.40–42.89)	58.05 (54.26–61.78)	2.32 (1.50–3.58)	46.32 (43.32–49.38)	53.95 (51.82–56.03)	1.36 (0.98–1.87)
No	37.99 (36.70–39.29)	44.68 (43.65–45.72)	1.32 (1.13–1.54)	33.95 (31.87–36.08)	51.04 (49.26–52.84)	2.02 (1.61–2.55)	36.92 (35.82–38.03)	46.29 (45.40–47.19)	1.47 (1.29–1.67)
No need for health care	47.46 (44.66–50.23)	47.02 (44.59–49.48)	0.98 (0.69–1.40)	29.25 (25.73–33.08)	42.69 (39.29–46.16)	1.80 (1.10–2.95)	41.58 (39.33–43.86)	45.58 (43.60–47.59)	1.18 (0.88–1.58)
Unmet need for health care due to inability to afford prescribed medicines*⁣*^*∗∗*^^a,c,d,e^									
Yes	49.05 (45.23–52.82)	48.66 (46.01–51.33)	0.98 (0.62–1.55)	47.16 (41.73–52.66)	50.60 (46.38–54.69)	1.15 (0.69–1.90)	48.44 (45.36–51.60)	49.22 (46.97–51.46)	1.03 (0.73–1.46)
No	38.74 (37.48–39.99)	45.53 (44.53–46.53)	1.32 (1.14–1.53)	31.44 (29.45–33.47)	51.93 (50.24–53.64)	2.35 (1.89–2.93)	36.82 (35.76–37.90)	47.18 (46.32–48.04)	1.53 (1.35–1.73)
No need for health care	47.92 (44.71–51.07)	47.64 (44.74–50.67)	0.99 (0.65–1.52)	32.70 (28.7–37.06)	43.58 (39.58–47.62)	1.59 (0.93–2.71)	42.78 (40.24–45.37)	46.22 (43.84–48.62)	1.15 (0.82–1.61)
Unmet need for health care due to inability to afford mental health care*⁣*^*∗∗*^^a,b,d,e,f^									
Yes	45.46 (42.05–48.85)	54.55 (51.83–57.18)	1.44 (0.95–2.18)	41.24 (33.68–49.32)	60.49 (54.81–66.27)	2.18 (1.09–4.34)	44.81 (41.69–47.94)	55.58 (53.13–57.99)	1.54 (1.06–2.22)
No	39.90 (38.52–41.27)	45.14 (44.05–46.24)	1.24 (1.05–1.46)	32.92 (30.48–35.36)	51.92 (49.82–54.05)	2.20 (1.65–2.94)	38.32 (37.13–39.53)	46.58 (45.61–47.56)	1.40 (1.21–1.62)
No need for health care	39.98 (37.65–42.34)	44.88 (43.00–46.75)	1.22 (0.94–1.59)	32.94 (30.35–35.50)	48.31 (46.16–50.52)	1.90 (1.45–2.49)	36.93 (35.19–38.67)	46.34 (44.92–47.76)	1.47 (1.22–1.78)

*Note:* European Health Interview Survey Wave 3 (2018–2020). *⁣*^*∗*^ = according to the PHQ-8; *⁣*^*∗∗*^ = in the past 12 months.

Abbreviation: OR, odds ratio.

^a^Statistically significant self-medication differences in men with recognized depression.

^b^Statistically significant self-medication differences in women with recognized depressed.

^c^Statistically significant self-medication differences in men with depression symptoms.

^d^Statistically significant self-medication differences in women with depression symptoms.

^e^Statistically significant self-medication differences in men for both groups.

^f^Statistically significant self-medication differences in women for both groups.

**Table 4:** Multivariable analysis of self-medication in depressed and noninstitutionalized residents aged 15 and over in the European Union according to demographic, socioeconomic, lifestyle, and health variables.

Independent variables	Recognized depression			Depression symptoms*⁣*^*∗*^			Both groups		
	Male	Female	Both sexes	Male	Female	Both sexes	Male	Female	Both sexes
Sex (ref. male)									
Female	—	—	1.41 (1.22–1.64)	—	—	2.25 (1.86–2.71)	—	—	1.37 (1.18–1.60)
Depression diagnosis tool (ref. recognized depression)									
Depression symptoms	—	—	—	—	—	—	0.69 (0.56–0.84)	1.28 (1.11–1.48)	0.70 (0.57–0.86)
Women with depression symptoms (ref. men with recognized depression)	—	—	—	—	—	—	—	—	1.75 (1.36–2.26)
Age (ref. 75+ years)									
15–24 years	1.16 (0.67–2.02)	1.47 (0.98–2.18)	1.15 (0.82–1.60)	0.77 (0.43–1.37)	1.60 (1.02–2.51)	1.15 (0.80–1.64)	1.01 (0.67–1.53)	1.50 (1.09–2.07)	1.06 (0.81–1.39)
25–44 years	1.00 (0.65–1.54)	1.35 (1.01–1.81)	1.12 (0.86–1.46)	1.17 (0.72–1.92)	1.93 (1.33–2.78)	1.38 (0.98–1.95)	1.07 (0.78–1.46)	1.47 (1.15–1.89)	1.15 (0.93–1.43)
45–64 years	0.89 (0.59–1.35)	1.02 (0.81–1.27)	0.95 (0.76–1.19)	0.72 (0.48–1.10)	1.45 (1.08–1.95)	0.99 (0.77–1.29)	0.86 (0.64–1.16)	1.07 (0.89–1.29)	0.94 (0.79–1.12)
65–74 years	0.80 (0.50–1.28)	1.04 (0.83–1.31)	0.95 (0.76–1.20)	1.34 (0.89–2.03)	1.08 (0.79–1.47)	1.20 (0.93–1.54)	0.98 (0.70–1.36)	1.06 (0.88–1.28)	1.00 (0.84–1.20)
Degree of urbanization (ref. rural areas)									
Cities	—	—	—	1.37 (1.00–1.88)	—	—	—	—	—
Towns and suburbs	—	—	—	1.00 (0.70–1.45)	—	—	—	—	—
Education level (ref. no formal education)									
Primary school	—	—	—	—	1.92 (1.21–3.05)	1.51 (1.00–2.29)	0.93 (0.56–1.53)	2.75 (2.10–3.61)	2.06 (1.57–2.70)
Secondary school	—	—	—	—	2.84 (1.78–4.55)	2.32 (1.52–3.54)	1.50 (0.93–2.42)	4.66 (3.53–6.14)	3.25 (2.48–4.25)
Higher education	—	—	—	—	3.37 (2.04–5.57)	2.79 (1.78–4.37)	1.67 (1.02–2.71)	5.63 (4.20–7.54)	3.74 (2.83–4.93)
Employment status (ref. unemployed)									
Employed	—	1.52 (1.06–2.18)	1.33 (1.00–1.77)	2.86 (1.57–5.22)	—	1.65 (0.98–2.76)	—	1.52 (1.09–2.11)	1.45 (1.12–1.88)
Inactive	—	1.28 (0.90–1.83)	1.20 (0.89–1.61)	1.26 (0.69–2.29)	—	1.05 (0.62–1.76)	—	1.23 (0.89–1.72)	1.18 (0.90–1.54)
Income level (ref. 1st quintile)									
2nd quintile	—	—	—	1.09 (0.71–1.65)	—	—	—	—	—
3rd quintile	—	—	—	1.30 (0.79–2.14)	—	—	—	—	—
4th quintile	—	—	—	1.70 (1.10–2.62)	—	—	—	—	—
5th quintile	—	—	—	1.59 (0.99–2.57)	—	—	—	—	—
Alcohol consumption (ref. once a month or less)									
More than once a month	1.42 (1.12–1.81)	—	1.20 (1.03–1.40)	—	—	—	1.36 (1.11–1.66)	—	1.16 (1.02–1.33)
Physical activity (ref. low physical activity)									
Moderate physical activity	1.27 (0.94–1.72)	1.22 (1.00–1.48)	1.22 (1.02–1.46)	1.59 (1.05–2.41)	1.01 (0.72–1.4)	1.09 (0.83–1.44)	1.26 (0.98–1.63)	1.15 (0.97–1.37)	1.14 (0.98–1.34)
High physical activity	1.30 (0.99–1.71)	1.50 (1.21–1.87)	1.39 (1.17–1.66)	1.53 (1.05–2.22)	1.43 (1.02–2.00)	1.37 (1.05–1.78)	1.32 (1.05–1.66)	1.50 (1.25–1.81)	1.38 (1.18–1.60)
Medication availability cluster (ref. pharmacy-only)									
Nonpharmacy	2.23 (1.82–2.72)	1.55 (1.37–1.76)	2.10 (1.86–2.36)	0.64 (0.48–0.86)	0.71 (0.59–0.87)	0.69 (0.59–0.82)	1.89 (1.59–2.25)	1.42 (1.27–1.59)	1.98 (1.78–2.21)
Limited pharmacy	1.43 (1.12–1.84)	1.09 (0.92–1.28)	1.32 (1.15–1.53)	0.38 (0.27–0.54)	0.52 (0.40–0.66)	0.46 (0.38–0.57)	1.17 (0.95–1.44)	0.95 (0.82–1.10)	1.15 (1.02–1.31)
Long-standing health problem (ref. no)									
Yes	—	1.35 (1.07–1.70)	—	—	—	—	—	—	—
Use of prescribed medicine in the past 2 weeks (ref. no)									
Yes	—	—	—	1.68 (1.12–2.52)	—	—	—	1.27 (1.04–1.55)	1.02 (0.85–1.21)
Visit to a medical or surgical specialist*⁣*^*∗∗*^ (ref. no)									
Yes	—	1.21 (1.01–1.45)	1.24 (1.05–1.46)	—	1.65 (1.30–2.08)	1.49 (1.23–1.81)	—	1.32 (1.13–1.53)	1.34 (1.17–1.54)
Unmet need for health care due to distance or transportation problems*⁣*^*∗∗*^ (ref. no need for health care)									
Yes	1.91 (1.06–3.46)	—	1.50 (1.05–2.15)	—	—	—	1.89 (1.18–3.04)	1.55 (1.20–1.99)	1.35 (0.99–1.85)
No	0.88 (0.63–1.25)	—	0.84 (0.67–1.06)	—	—	—	0.97 (0.73–1.28)	1.06 (0.87–1.29)	0.88 (0.72–1.09)
Unmet need for health due to inability to afford medical examination or treatment*⁣*^*∗∗*^ (ref. no need for health care)									
Yes	—	—	—	—	2.02 (1.32–3.08)	1.97 (1.40–2.77)	—	—	1.38 (1.07–1.79)
No	—	—	—	—	1.47 (1.06–2.03)	1.42 (1.09–1.86)	—	—	1.00 (0.82–1.22)

*Note:* Inactive, retirees, students, and those performing domestic tasks, carrying out compulsory service, or unable to work for health reasons. 1st quintile contains the lowest values and the 5th quintile the highest values. European Health Interview Survey Wave 3 (2018–2020). *⁣*^*∗*^ = according to the PHQ-8; *⁣*^*∗∗*^ = in the past 12 months;

## Data Availability

Data requests must be made to Eurostat. Instructions for requesting data access can be found at https://ec.europa.eu/eurostat/web/microdata/european-health-interview-survey. The code for data processing is available upon request to the authors.

## Supporting Information

Supporting Information 1 Table A. Self-medication prevalence by country and sex.

Supporting Information 2 STROBE Checklist.
