# Supplementary material for: Self-Medication in Individuals With Depression and Symptoms of Depression in the European Union: Prevalence and Associated Factors
Source: Depress Anxiety. 2025 Aug 15;2025:4661541. doi: 10.1155/da/4661541 (PMC12373476; doi:10.1155/da/4661541)
Supplement: Supporting Information 1 — Table A. Self-medication prevalence by country and sex. [file 4661541.f1.pdf]

Supplementary Table A. Self-medication prevalence by country and sex in depressed, non-institutionalized residents aged 15 and over in the European Union. European Health Interview Survey Wave 3 (2018-2020)

	Recognized Depression			Depression Symptoms*			Both Groups		
	**Male**	**Female**	**OR Female**	**Male**	**Female**	**OR Female**	**Male**	**Female**	**OR Female**
**Austria**	40.66 (34.06-47.36)	54.53 (49.34-59.73)	1.75 (1.28-2.40)	31.40 (21.90-41.93)	51.34 (42.73-60.58)	2.30 (1.33-3.97)	38.09 (32.51-43.69)	53.73 (49.32-58.33)	1.88 (1.44-2.47)
**Belgium**	30.58 (24.73-36.76)	35.44 (30.84-40.32)	1.25 (0.77-2.02)	31.60 (24.32-39.06)	35.92 (30.10-42.06)	1.21 (0.67-2.19)	30.99 (26.38-35.74)	35.63 (31.99-39.43)	1.23 (0.85-1.79)
**Bulgaria**	40.77 (26.67-54.57)	59.45 (50.12-68.94)	2.13 (1.12-4.02)	51.32 (39.34-63.74)	62.10 (53.91-70.53)	1.55 (0.93-2.58)	46.91 (37.47-56.16)	60.92 (54.46-67.01)	1.76 (1.19-2.61)
**Cyprus**	66.32 (34.80-86.27)	74.24 (53.58-89.76)	1.46 (0.82-2.60)	79.65 (6.08-93.92)	69.75 (17.67-96.13)	0.59 (0.12-2.89)	68.19 (38.76-87.55)	73.71 (53.92-88.31)	1.31 (0.76-2.24)
**Czechia**	57.40 (48.96-65.44)	71.78 (65.96-77.46)	1.89 (1.14-3.13)	60.15 (47.65-72.70)	75.42 (64.33-83.27)	2.03 (0.93-4.41)	58.20 (51.28-65.14)	72.70 (67.52-77.40)	1.91 (1.25-2.92)
**Germany**	41.91 (40.28-43.55)	48.35 (46.93-49.77)	1.29 (0.98-1.70)	25.54 (22.96-28.23)	49.83 (47.15-52.55)	2.89 (1.69-4.97)	38.13 (36.71-39.54)	48.67 (47.42-49.93)	1.53 (1.21-1.95)
**Denmark**	50.09 (43.09-56.45)	59.80 (53.79-65.68)	1.48 (1.03-2.11)	46.67 (36.42-56.98)	66.47 (58.52-74.12)	2.26 (1.32-3.89)	49.09 (43.38-54.64)	62.12 (57.37-66.88)	1.70 (1.26-2.29)
**Estonia**	61.05 (42.44-78.22)	79.79 (65.78-87.96)	2.52 (1.45-4.35)	62.62 (40.58-77.25)	75.20 (61.86-87.05)	1.80 (1.04-3.16)	61.82 (47.09-73.29)	77.70 (68.17-85.00)	2.15 (1.45-3.17)
**Greece**	27.55 (19.27-38.00)	29.77 (23.81-36.27)	1.11 (0.58-2.13)	29.76 (16.94-45.56)	31.21 (19.60-47.84)	1.07 (0.37-3.05)	28.22 (21.05-36.86)	30.01 (24.71-36.16)	1.09 (0.62-1.90)
**Spain****	13.66 (11.14-16.57)	17.07 (15.20-19.12)	1.30 (0.84-1.99)	N/A	N/A	N/A	N/A	N/A	N/A
**Finland**	75.65 (67.94-82.46)	86.89 (81.64-91.03)	2.13 (1.16-3.89)	66.76 (52.35-76.98)	79.86 (70.25-87.26)	1.97 (0.91-4.29)	73.04 (66.43-79.10)	84.80 (80.21-88.58)	2.05 (1.28-3.30)
**Croatia**	40.03 (31.62-48.22)	47.67 (41.55-53.78)	1.36 (0.89-2.08)	49.25 (32.68-64.69)	57.64 (46.93-67.21)	1.40 (0.67-2.90)	41.97 (34.49-49.43)	50.26 (44.87-55.42)	1.40 (0.97-2.01)
**Hungary**	66.95 (57.91-75.16)	67.81 (61.46-73.88)	1.04 (0.55-1.95)	54.28 (45.22-63.96)	63.72 (56.91-70.62)	1.47 (0.82-2.68)	60.79 (54.16-67.02)	65.91 (61.09-70.35)	1.25 (0.81-1.91)
**Ireland**	41.31 (30.14-53.67)	32.22 (22.60-42.14)	0.67 (0.37-1.23)	32.07 (19.52-45.16)	41.94 (31.14-53.27)	1.53 (0.77-3.05)	37.40 (28.67-46.31)	36.74 (29.29-44.15)	0.97 (0.61-1.53)
**Italy**	19.15 (14.95-24.24)	26.23 (22.70-29.91)	1.50 (1.13-2.00)	20.64 (14.75-27.36)	21.68 (17.06-26.70)	1.06 (0.71-1.59)	19.68 (16.17-23.67)	24.74 (21.86-27.66)	1.34 (1.06-1.69)
**Lithuania**	60.60 (47.26-73.91)	76.24 (67.70-83.71)	2.08 (1.24-3.50)	64.38 (44.49-80.47)	72.60 (60.05-84.88)	1.46 (0.68-3.12)	61.88 (50.82-72.57)	75.13 (67.69-81.37)	1.86 (1.21-2.85)
**Luxembourg**	45.53 (23.74-63.79)	43.10 (26.89-60.99)	0.90 (0.60-1.37)	30.85 (9.27-60.58)	43.92 (23.74-66.78)	1.76 (0.97-3.19)	40.68 (25.92-59.38)	43.41 (30.42-57.81)	1.11 (0.80-1.56)
**Latvia**	51.70 (37.05-67.83)	60.97 (49.93-70.31)	1.46 (0.93-2.28)	29.09 (08.35-56.51)	57.65 (38.87-74.02)	3.31 (1.48-7.39)	46.52 (32.74-59.71)	60.16 (50.49-68.32)	1.73 (1.17-2.56)
**Malta**	38.46 (7.68-71.36)	54.80 (25.41-82.70)	1.94 (0.94-4.00)	43.40 (12.28-87.72)	44.18 (13.89-76.55)	1.03 (0.43-2.49)	40.45 (15.31-69.63)	50.17 (27.22-72.78)	1.48 (0.85-2.58)
**Netherlands**	50.29 (46.07-54.71)	59.51 (55.76-63.15)	1.45 (1.03-2.03)	33.81 (28.08-40.10)	57.37 (52.69-62.18)	2.63 (1.64-4.22)	45.09 (41.59-48.70)	58.69 (55.73-61.58)	1.73 (1.32-2.26)
**Poland**	54.33 (49.28-59.54)	61.45 (57.94-64.92)	1.34 (0.91-1.97)	41.00 (34.42-48.08)	58.10 (53.74-62.52)	1.99 (1.17-3.14)	49.61 (45.41-53.70)	60.13 (57.37-62.84)	1.53 (1.14-2.06)
**Portugal**	23.11 (18.74-28.17)	25.74 (22.78-28.96)	1.15 (0.77-1.72)	24.69 (17.40-33.46)	30.41 (24.67-37.10)	1.33 (0.67-2.65)	23.52 (19.72-27.87)	26.74 (24.01-29.55)	1.19 (0.84-1.68)
**Romania**	18.38 (9.94-30.37)	16.49 (10.66-24.75)	0.88 (0.37-2.09)	27.30 (22.07-32.91)	33.62 (29.15-38.64)	1.34 (0.93-1.96)	25.74 (21.11-30.79)	29.86 (25.90-34.01)	1.23 (0.87-1.73)
**Sweden**	46.55 (41.96-51.45)	55.52 (51.35-59.51)	1.43 (1.11-1.84)	36.82 (29.88-44.37)	54.17 (47.86-60.51)	2.02 (1.36-3.03)	43.78 (39.76-47.75)	55.12 (51.65-58.51)	1.58 (1.27-1.95)
**Slovenia**	36.60 (23.59-50.40)	48.51 (37.49-58.93)	1.63 (1.15-2.31)	36.27 (21.98-52.38)	48.35 (33.89-62.02)	1.64 (1.09-2.48)	36.46 (26.42-46.81)	48.45 (39.47-56.68)	1.63 (1.26-2.13)
**Slovakia**	52.22 (41.08-64.72)	76.77 (68.76-83.12)	3.02 (1.65-5.54)	65.10 (42.62-79.56)	67.67 (51.25-78.55)	1.12 (0.43-2.88)	55.61 (45.25-65.51)	74.51 (68.06-80.84)	2.33 (1.40-3.88)
**Total**	40.52 (39.42-41.62)	45.68 (44.80-46.56)	1.23 (1.08-1.41)	33.25 (31.59-34.95)	49.87 (48.45-51.30)	1.99 (1.66-2.39)	38.46 (37.54-39.39)	46.84 (46.09-47.58)	1.41 (1.27-1.57)

* = according to the PHQ-8; ** = PHQ variable questions excluded from dataset; OR = odds ratio; N/A = not available
