# Supplementary material for: Self-Medication in Individuals With Depression and Symptoms of Depression in the European Union: Prevalence and Associated Factors
Source: Depress Anxiety. 2025 Aug 15;2025:4661541. doi: 10.1155/da/4661541 (PMC12373476; doi:10.1155/da/4661541)
Supplement: Supporting Information 2 — STROBE Checklist. [file 4661541.f2.pdf]

STROBE Statement—Checklist of items that should be included in reports of ***cross-sectional studies***

	**Item No**	**Recommendation**
**Title and abstract**	1	(*a*) Indicate the study's design with a commonly used term in the title or the abstract<br>"Cross-sectional" featured in the abstract on page 1.
		(*b*) Provide in the abstract an informative and balanced summary of what was done and what was found<br>Featured in the abstract on page 1.
**Introduction**		
Background/rationale	2	Explain the scientific background and rationale for the investigation being reported<br>Featured in the introduction on pages 1 and 2.
Objectives	3	State specific objectives, including any prespecified hypotheses<br>Featured in the introduction on page 2.
**Methods**		
Study design	4	Present key elements of study design early in the paper<br>Featured in materials and methods on pages 2 and 3.
Setting	5	Describe the setting, locations, and relevant dates, including periods of recruitment, exposure, follow-up, and data collection<br>Featured in materials and methods on pages 2 and 3.
Participants	6	(*a*) Give the eligibility criteria, and the sources and methods of selection of participants<br>Featured in materials and methods on page 2.
Variables	7	Clearly define all outcomes, exposures, predictors, potential confounders, and effect modifiers. Give diagnostic criteria, if applicable<br>Featured in materials and methods on pages 2 and 3.
Data sources/ measurement	8*	For each variable of interest, give sources of data and details of methods of assessment (measurement). Describe comparability of assessment methods if there is more than one group.<br>Featured in materials and methods on pages 2 and 3.
Bias	9	Describe any efforts to address potential sources of bias<br>Featured in materials and methods on pages 2 and 3, and in the discussion on pages 7 and 8.
Study size	10	Explain how the study size was arrived at<br>Featured in materials and methods on page 2.
Quantitative variables	11	Explain how quantitative variables were handled in the analyses. If applicable, describe which groupings were chosen and why<br>Featured in materials and methods on page 2 and 3.
Statistical methods	12	(*a*) Describe all statistical methods, including those used to control for confounding<br>Featured in materials and methods on page 3.
		(*b*) Describe any methods used to examine subgroups and interactions<br>Featured in materials and methods on pages 2 and 3.
		(*c*) Explain how missing data were addressed<br>Featured in materials and methods on page 2.
		(*d*) If applicable, describe analytical methods taking account of sampling strategy<br>Not applicable.
		(*e*) Describe any sensitivity analyses<br>Featured in materials and methods on page 3.

**Results**		
Participants	13*	(a) Report numbers of individuals at each stage of study—eg numbers potentially eligible, examined for eligibility, confirmed eligible, included in the study, completing follow-up, and analysed<br>Not applicable.
		(b) Give reasons for non-participation at each stage<br>Not applicable.
		(c) Consider use of a flow diagram<br>Not applicable.
Descriptive data	14*	(a) Give characteristics of study participants (eg demographic, clinical, social) and information on exposures and potential confounders<br>Featured in results and table 1 on pages 3, 4, 13, and 14.
		(b) Indicate number of participants with missing data for each variable of interest<br>Not applicable.
Outcome data	15*	Report numbers of outcome events or summary measures<br>Not applicable.
Main results	16	(*a*) Give unadjusted estimates and, if applicable, confounder-adjusted estimates and their precision (eg, 95% confidence interval). Make clear which confounders were adjusted for and why they were included<br>Featured in results on pages 3 and 4, and tables 1, 2, and 3 on pages 15-20.
		(*b*) Report category boundaries when continuous variables were categorized<br>Featured in materials and methods on pages 2 and 3.
		(*c*) If relevant, consider translating estimates of relative risk into absolute risk for a meaningful time period<br>Not applicable.
Other analyses	17	Report other analyses done—eg analyses of subgroups and interactions, and sensitivity analyses<br>Reported in results on pages 3 and 4.
**Discussion**		
Key results	18	Summarise key results with reference to study objectives<br>Featured in the discussion on pages 6, 7, and 8.
Limitations	19	Discuss limitations of the study, taking into account sources of potential bias or imprecision. Discuss both direction and magnitude of any potential bias<br>Featured in the discussion on pages 7 and 8.
Interpretation	20	Give a cautious overall interpretation of results considering objectives, limitations, multiplicity of analyses, results from similar studies, and other relevant evidence<br>Featured in the discussion on pages 6, 7, and 8.
Generalisability	21	Discuss the generalisability (external validity) of the study results<br>Featured in the discussion on pages 6, 7, and 8.
**Other information**		
Funding	22	Give the source of funding and the role of the funders for the present study and, if applicable, for the original study on which the present article is based<br>Featured in statements & declarations on page 7.

*Give information separately for exposed and unexposed groups.

Note: An Explanation and Elaboration article discusses each checklist item and gives methodological background and published examples of transparent reporting. The STROBE checklist is best used in conjunction with this article (freely available on the Web sites of PLoS Medicine at http://www.plosmedicine.org/, Annals of Internal Medicine at http://www.annals.org/, and Epidemiology at http://www.epidem.com/). Information on the STROBE Initiative is available at www.strobe-statement.org.
